# Prediction model of preeclampsia using machine learning based methods: a population based cohort study in China

**DOI:** 10.3389/fendo.2024.1345573

**Published:** 2024-06-11

**Authors:** Taishun Li, Mingyang Xu, Yuan Wang, Ya Wang, Huirong Tang, Honglei Duan, Guangfeng Zhao, Mingming Zheng, Yali Hu

**Affiliations:** ^1^ Department of Obstetrics and Gynecology, Nanjing Drum Tower Hospital, The Affiliated Hospital of Nanjing University Medical School, Nanjing, China; ^2^ Medical Statistics and Analysis Center, Nanjing Drum Tower Hospital, The Affiliated Hospital of Nanjing University Medical School, Nanjing, China; ^3^ Information Management Division, Nanjing Drum Tower Hospital, The Affiliated Hospital of Nanjing University Medical School, Nanjing, China

**Keywords:** preeclampsia, machine learning, cohort, voting classifier, competing risk model

## Abstract

**Introduction:**

Preeclampsia is a disease with an unknown pathogenesis and is one of the leading causes of maternal and perinatal morbidity. At present, early identification of high-risk groups for preeclampsia and timely intervention with aspirin is an effective preventive method against preeclampsia. This study aims to develop a robust and effective preeclampsia prediction model with good performance by machine learning algorithms based on maternal characteristics, biophysical and biochemical markers at 11–13 + ^6^ weeks’ gestation, providing an effective tool for early screening and prediction of preeclampsia.

**Methods:**

This study included 5116 singleton pregnant women who underwent PE screening and fetal aneuploidy from a prospective cohort longitudinal study in China. Maternal characteristics (such as maternal age, height, pre-pregnancy weight), past medical history, mean arterial pressure, uterine artery pulsatility index, pregnancy-associated plasma protein A, and placental growth factor were collected as the covariates for the preeclampsia prediction model. Five classification algorithms including Logistic Regression, Extra Trees Classifier, Voting Classifier, Gaussian Process Classifier and Stacking Classifier were applied for the prediction model development. Five-fold cross-validation with an 8:2 train-test split was applied for model validation.

**Results:**

We ultimately included 49 cases of preterm preeclampsia and 161 cases of term preeclampsia from the 4644 pregnant women data in the final analysis. Compared with other prediction algorithms, the AUC and detection rate at 10% FPR of the Voting Classifier algorithm showed better performance in the prediction of preterm preeclampsia (AUC=0.884, DR at 10%FPR=0.625) under all covariates included. However, its performance was similar to that of other model algorithms in all PE and term PE prediction. In the prediction of all preeclampsia, the contribution of PLGF was higher than PAPP-A (11.9% VS 8.7%), while the situation was opposite in the prediction of preterm preeclampsia (7.2% VS 16.5%). The performance for preeclampsia or preterm preeclampsia using machine learning algorithms was similar to that achieved by the fetal medicine foundation competing risk model under the same predictive factors (AUCs of 0.797 and 0.856 for PE and preterm PE, respectively).

**Conclusions:**

Our models provide an accessible tool for large-scale population screening and prediction of preeclampsia, which helps reduce the disease burden and improve maternal and fetal outcomes.

## 1 Introduction

Pre-eclampsia (PE) is one of the great obstetrical syndromes (1–3) and affects 2–5% of pregnancies worldwide. PE is a major cause of maternal and perinatal morbidity and mortality (4, 5), accounting for 70000 maternal deaths and 500000 fetal deaths worldwide every year (6). The pathogenesis of PE remains unclear and curative treatments are limited in clinical practice, with placental ischemia, endothelial dysfunction, and immune maladaptation being possible mechanisms leading to PE (7–9). Previous researches showed that early intervention with aspirin given from 12 gestational weeks can effectively reduce the occurrence of PE (10, 11). Thus, accurately predicting and identifying high-risk groups of PE during the first trimester of pregnancy is beneficial for timely prevention strategies and improving maternal and fetal outcomes. The prevailing strategy for PE screening involves identifying risk factors based on maternal demographic characteristics and medical history (12, 13). As outlined in the guidelines from the American College of Obstetricians and Gynecologists (ACOG), Obstetrics and Gynecology branch of the Chinese Medical Association, if the pregnant women exhibit any high-risk factors (like the history of PE, chronic hypertension, renal disease, type 1 or 2 diabetes and autoimmune disease etc.) or if they have at least two moderate-risk factors (such as age≥40 years, nulliparity, etc.) they should take asplin (14, 15). An increasing body of evidence suggests that the incorporation of maternal history with some physical signs, such as mean arterial pressure (MAP), uterine artery pulsatility index (UtA-PI), and biomarkers such as serum pregnancy-associated plasma protein A (PAPP-A), and serum placental growth factor (PLGF) will improve the prediction efficiency of PE. The Competing risks model in screening for PE maternal characteristics and medical history established by wright indicates that the model-based Bayes theorem using the combination of *a priori* risk from maternal characteristics and the biomarkers results greatly improved the overall screening performance of PE (16, 17).

Recently, Machine Learning (ML), a subset of artificial intelligence, has emerged as a revolutionary tool in the realm of complex diseases prediction and diagnosis (18, 19). With the capacity to process vast amounts of data and extract meaningful patterns, ML algorithms have been instrumental in early disease detection, enhancing diagnostic accuracy, and offering insights beyond the capabilities of traditional methods. Ansbacher-Feldman Z (19) and Gil MM (20) research have found that machine-learning models utilizing neural networks can effectively screen for PE with high accuracy, using maternal characteristics and raw biomarker data. Melinte-Popescu AS’s study (21) included four machine learning-based models: decision tree (DT), naïve Bayes (NB), support vector machine (SVM), and random forest (RF) for PE screening in the first trimester, the study indicates that machine learning-based models could be useful tools for PE prediction in the first trimester of pregnancy. Torres-Torres J (22) study also finds that elastic net regression offers a potential solution for developing accurate and efficient prediction models for PE and offers significant clinical benefits. The predictive model performance of PE may vary among different ethnic groups. Currently, there are few studies on developing machine learning algorithm-based prediction models specifically for Chinese population cohorts. Liu M study (23) shows that machine learning, particularly using RF, accurately predicts PE by using clinical history and prenatal screening results in a retrospective cohort study in China.

In this study, we develop predictive models for PE using new ML techniques such as the Extra Trees Classifier (ETC), Voting Classifier (VC), Gaussian Process Classifier (GPC), and Stacking Classifier (SC) based on prospective cohort study in China. These advanced algorithms offer more nuanced and potentially more accurate predictive capabilities compared to traditional machine learning algorithms.

## 2 Materials and methods

### 2.1 Study population

This was a prospective cohort longitudinal study from early pregnancy within 14 weeks of gestation (with a crown-rump length of 45–84 mm) to childbirth for PE studybased on the combined screening for fetal aneuploidy in early pregnancy. This study included 5116 singleton pregnant women who underwent PE screening and fetal aneuploidy at the affiliated Nanjing Drum Tower Hospital of Nanjing University Medical School from January 2017 to September 2020. This study excluded patients with incomplete information (including 275 missing PLGF data and 34 missing UtA-PI data), and those who experienced natural miscarriages before 28 weeks (56 cases). Also, patients who terminated their pregnancy for personal reasons (6 cases), those who terminated due to fetal malformations or chromosomal abnormalities (18 cases), and those lost to follow-up (83 cases) were excluded. A total of 4,644 participants were ultimately included in this analysis. This study protocol was approved by the institutional review board of Nanjing Drum Tower Hospital (2016–113–01). This study followed the TRIPOD statement for reporting (24).

### 2.2 Model covariates and outcome

Covariates in the prediction model included (1) maternal demographic characteristics (maternal age, height, pre-pregnancy weight, nulliparous, method of conception [natural; ovulation induction; *in-vitro* fertilization-embryo transfer], family history of PE, and smoking); (2) past medical history (history of PE, history of chronic hypertension, history of chronic kidney disease, type 1 or type 2 diabetes, history of systemic lupus erythematosus and/or antiphospholipid syndrome); (3) Biophysical markers (MAP, UtA-PI); (4) Biochemical markers (PAPP-A and PLGF). The measurement of biophysical and biochemical markers was conducted between 11 weeks and 13 + ^6^ weeks of gestation.

The reasons for choosing four biophysical or biochemical markers as predictive factors are as follows: UtA-PI is a measure of the resistance to blood flow in the uterine arteries. High resistance (high UtA-PI) suggests poor placentation, as it reflects the inadequate remodeling of spiral arteries. The uterine blood supply consists of a vascular structure decreasing in size as it progresses through the myometrium and endometrium, culminating in spiral arteries. During early pregnancy, >100 spiral arteries are remodeled into high-flow uteroplacental vessels with low resistance, to ensure the provision of an adequate blood supply to the developing fetus. These vascular changes are therefore crucial for decreasing maternal vascular resistance and increasing uteroplacental blood flow by up to ten-fold during this time (from ~50 ml per minute pre-pregnancy to ~500 ml per minute upon completion of placentation). Impaired or incomplete spiral artery remodeling is implicated in PE, intrauterine growth restriction and recurrent miscarriage, due to various degrees of insufficient blood flow to the fetus. In PE pregnancies, abnormal spiral artery remodeling with incomplete placentation and poor placental perfusion, can lead to maternal systemic hypoxia and hypertensive pathology, activation of the maternal renal and cardiovascular systems with endothelial damage, and potential end-organ damage (25–28). MAP is a composite measure of cardiac output and systemic vascular resistance. Systemic vascular resistance is increased due to endothelial dysfunction in PE, which can be reflected in elevated MAP early in pregnancy before clinical symptoms appear (29); PAPP-A is a protein produced by the placenta. Low levels in the first trimester have been associated with poor placental development and function, leading to increased risk of PE (30); PLGF is an angiogenic factor that promotes placental blood vessel development. Low levels of PLGF are indicative of placental insufficiency and have been linked to the development of PE (31). The outcome in this study was the development of PE, PE is defined as the occurrence of a systolic blood pressure of ≥140 mmHg and/or a diastolic blood pressure of ≥90 mmHg in pregnant women after 20 weeks of gestation, accompanied by any one of the following: a urinary protein quantitation of ≥0.3 g/24 h, a urine protein/creatinine ratio of ≥0.3, or a random urinary protein level of ≥ (+) (as a testing method when protein quantitation is not conducted unconditionally). We divided PE into preterm PE (delivery gestational week <37 weeks) and term PE (delivery gestational week ≥37 weeks) (32). Gestational week in this study was determined by measurements of fetal crown-rump length (CRL) within the first trimester of pregnancy (33).

### 2.3 Quality control

All selected pregnant women were interviewed on-site by researchers to collect their medical history. The pregnancy outcomes for women who gave birth in our hospital were obtained from medical records (accounting for 90.8%), while those for women who gave birth in other hospitals were collected through dedicated telephone follow-ups (accounting for 9.2%). All research data were collected with the Viewpoint 6.0 software by data administrators.

Quality control standards for the detections of biophysical and biochemical markers were as follows (1). MAP: Blood pressure was measured on-site in a standardized manner using an automatic blood pressure measuring device (3BTO-A2, Microlife Corporation, Taiwan, China) by trained designated doctors. The blood pressure monitor was regularly calibrated by the hospital’s quality inspection department. Before measuring blood pressure, pregnant women were seated comfortably for at least 5 minutes, and a cuff of appropriate size was selected based on the arm circumference. The blood pressure of both arms were measured simultaneously, recorded every 1 minute, until the difference in consecutive readings was within 10 mmHg for systolic pressure and within 6 mmHg for diastolic pressure. The MAP for both arms were calculated based on the average of the last two stable measurements. The final blood pressure was determined by the higher average MAP of the two arms (34); (2) UtA-PI: The measurement of UtA-PI was conducted using the Voluson E8 color Doppler ultrasound diagnostic device from GE, USA, with the probe models RAB6-D/0B and RAB4–8-D/OB, and a frequency of 4–6 MHz. The measurement was performed by ultrasound doctors who had participated in FMF ultrasound technology training and passed the uterine artery monitoring qualification certification. In accordance with FMF measurement standards, pulse wave Doppler was used to obtain three similar continuous waveforms from the ascending uterine artery at the level of the cervical internal. The UtA-PI of both sides was measured and the average UtA-PI was calculated (35); (3) PAPP-A: PAPP-A was derived from the records of serum screening for Down syndrome during early pregnancy in our hospital. It was detected using the AutoDELFIA 1235 automatic immune analysis system (time-resolved fluorescence immunoassay method, AutoDELFIA PAPP-A reagent kit, PerkinElmer Company, Finland); (4) PLGF: Blood samples from all subjects were collected on the day of enrollment and sent to the sample bank for centrifugation within 2 hours without anticoagulants. After drawing blood for PlGF, the sample was inverted at least 5 times, then the clot was allowed to sit at room temperature for about 30 minutes before centrifugation. After centrifugation, the serum was stored at -80°C. The PlGF value was measured using the AutoDELFIA 1235 automatic immune analysis system (time-resolved fluorescence immunoassay, PerkinElmer AutoDELFIA PlGF reagent kit, Finland) or Cobas e602 system (Roche Diagnostics, Germany). Quality control requirements stipulate that the coefficient of variation for measurements of quality control materials with different concentrations in each batch must be less than 5%; and the measurement range for quality control materials in each test should be within two standard deviations.

### 2.4 Model development

In our model, five classification algorithms including Logistic Regression (LR)[2], Extra Trees Classifier (ETC), Voting Classifier (VC), Gaussian Process Classifier (GPC)[3] and Stacking Classifier (SC) were applied. To enhance the overall accuracy and robustness of our model, we incorporated certain ensemble learning techniques [6], the VC and SC which are frequently deployed in algorithm competitions. As for VC, multiple models were trained on the same dataset, and their predictions were combined to make a final prediction. The VC can be used for classification problems, where each individual model is an estimator, and the final prediction is made by combining the predictions of all the classifiers using a voting strategy. For our model, we chose Random Forest (RF) and ETC as its estimators. The voting criteria we used was soft voting, which calculates the voting based on the estimators’ predicted probabilities.

For the SC, multiple models, known as base models, were trained on the same dataset. Instead of directly combining their predictions, these outputs served as input features for another model. This subsequent model, the ‘meta’ classifier or ‘meta learner’, is particularly important in the stacking approach. In our setup, we employed the GPC as the meta classifier. This model was trained to make the final prediction, informed by the outputs of the base models. The unique advantage of the SC is its capability to discern nuanced patterns and relationships in the predictions of individual base models. For our base models, we opted for Support Vector Machines (SVM), ETC, and GPC. By strategically leveraging these models through the SC, we aim to harness the individual strengths of each model, potentially surpassing the accuracy and consistency that any single model could achieve on its own.

Since our dataset was unbalanced with a 1:23 positive and negative weights, we set class weight of LR, ETC, and all the estimators of VC to balanced, this automatically adjusted the weights inversely proportional to their frequencies in the data. Before training, we normalized all the data in the range from 0 to 1, which could help improve the performance and stability of training.

To optimize the performance of each algorithmic model, we implemented Bayesian Optimization, conducting hyperparameter tuning across 20 repetitions of 5-fold cross-validation. The Area Under the Receiver Operating Characteristic (AUC-ROC) curve was chosen as the metric to evaluate the effectiveness of tuning for each model. Given the complex nature of voting classifiers and stacking classifiers, which, as ensemble learning techniques, amalgamate diverse models and thus exhibit a vast hyperparameter space, we strategically tuned the hyperparameters of each constituent estimator in isolation. Following this meticulous individual optimization, we then proceeded to integrate these finely tuned estimators.

### 2.5 Model evaluation

We evaluated the performance of our model using both discrimination and calibration metrics. We used the area under the receiver operating characteristic curve (AUC-ROC), sensitivity, specificity to evaluate the discrimination of our model. We also determined the cut-off value for classification using the Youden Index[8], which is a commonly used algorithm for calculating the optimal cut-off point. This algorithm determines the cut-off value when sensitivity = 1 - specificity in the ROC-curve. We evaluated the calibration of our model using the Brier score, calibration slope, and calibration intercept. The Brier score measures the accuracy of probabilistic predictions, while the calibration slope and intercept indicate the reliability of the predicted probabilities.

ML models are often considered “black boxes,” making it difficult to interpret their results. In our study, we introduced SHAP (SHapley Additive exPlanations) values[9] to break down our models and explain their predictions. We plotted a beeswarm chart and a pie chart for the VC model to analyze the distribution of the feature values and the contribution of each feature.

### 2.6 Model validation

To validate the performance of our model, we applied a 5-fold cross-validation with an 8:2 train-test split. We conducted 200 repetitions of cross-validation with random seeds for all the models individually. After each fold in every repetition, we recorded the performance measurements. Upon concluding 1000 evaluations (5 folds * 200 repetitions), we employed the bootstrapping method to calculate the 95% confidence interval of the aggregate results, thus providing a robust measure of the model’s reliability.

### 2.7 Statistical analysis

Our model was developed using Scikit-learn 1.2.0 (sklearn), a widely recognized open-source ML library in Python. To ensure rigorous and transparent evaluation, all performance metrics were presented as point estimates accompanied by 95% confidence intervals (CIs). This interval estimation approach was pivotal in comparing the performance of different models. The selection of the best model was grounded on its Area Under the Receiver Operating Characteristic (AUROC), ascertained from the validation set. For the statistical description of continuous metrics, we employed mean and standard deviation, whereas categorical data were characterized using frequencies and percentages.

### 2.8 Comparison to previous studies

We have also evaluated our optimal algorithm for predicting PE or Pre-term PE, contrasting it with algorithms from previous studies conducted over the past five years. Studies were selected based on the following criteria: (1) The document type was required to be an original article; (2) The model predictive factors were required to include maternal baseline and clinical biomarkers; (3) Clinical biomarkers were required to include at least two of PLGF, PAPP-A, UtA-PI and MAP; (4) Preterm PE was required to be defined as occurring before 37 weeks of gestation; (5) The compared algorithm was required to be the best-performing one in the study; (6) All predictive indicators was required to be obtained early in pregnancy (within 14 weeks of gestation). All selected publications needed to report the sample size, data sources, model algorithms, predictive factors used in the model, and model performance indicators.

## 3 Results

### 3.1 Study population characteristics

**Figure 1:**
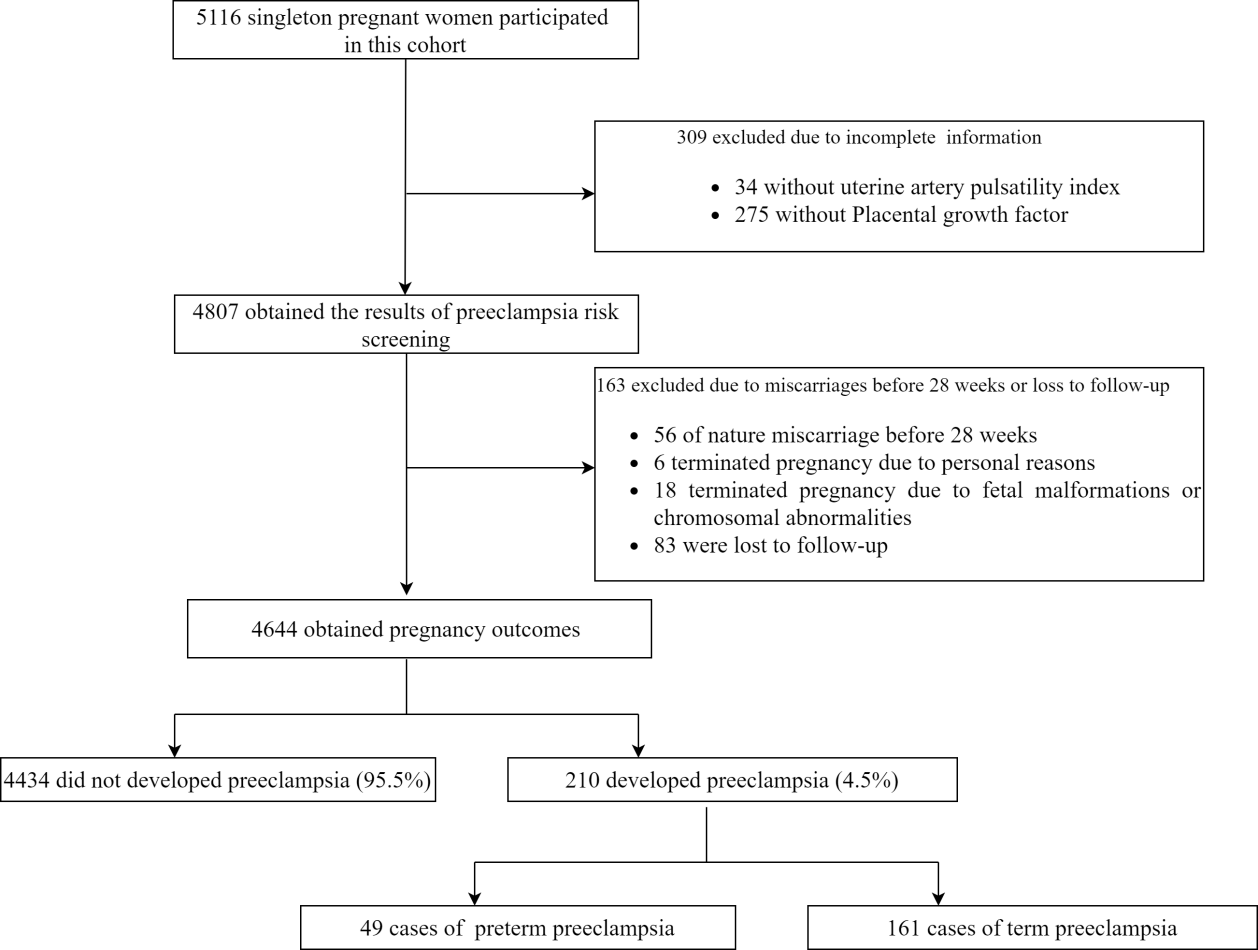
The flow chart of the study.

A total of 5116 singleton pregnant women participated in this cohort, 472 were excluded for various reasons (**Figure 1**), and 4644 were ultimately included for analysis. Among them, 210 pregnant women developed PE, with an incidence of 4.5%. This includes 49 cases of preterm PE and 161 cases of term PE. A detailed flow chart of the study is presented in **Figure 1**. **Table 1** shows maternal characteristics and biophysical markers for study subjects with and without PE (used in the prediction model of PE). Subjects who developed PE had older age and higher weight than those without PE. Participants with a history of PE, chronic hypertension, or a family history of PE were more likely to develop PE. The MAP in those who developed PE was higher than those who did not develop PE, but with lower level of PLGF and PAPP-A. There was statistically significant difference in maternal age, pre-pregnancy weight, nulliparous, history of diabetes mellitus, history of chronic hypertension, family history of PE, MAP, UtA-PI and PAPP-A between the preterm PE group and no preterm PE group.

**Table 1:** Study population characteristics.

Characteristic	Preeclampsia model			Preterm preeclampsia model		
	Preeclampsia (N=210)	No Preeclampsia (N=4434)	*P* value	Preterm preeclampsia (N=49)	No preterm preeclampsia (N=4595)	*P* value
Maternal age, year*	30.18 ± 3.74	29.63 ± 3.26	0.0379	31.02 ± 4.42	29.64 ± 3.27	0.0348
Height, cm*	162.00 ± 4.74	162.01 ± 4.78	0.9942	161.52 ± 4.23	162.01 ± 4.79	0.4737
Pre-pregnancy weight, kg*	62.36 ± 11.11	56.97 ± 8.50	<.0001	62.81 ± 10.66	57.15 ± 8.67	<.0001
Nulliparous, n (%)	171 (81.43)	3439 (77.56)	0.1879	31 (63.27)	3579 (77.89)	0.0144
Previous History of PE, n (%)	19 (9.05)	28 (0.63)	<.0001	12 (24.49)	35 (0.76)	<.0001
History of Diabetes Mellitus, n (%)	6 (2.86)	27 (0.61)	0.0032	2 (4.08)	31 (0.67)	0.0468
History of Chronic Hypertension, n (%)	30 (14.29)	25 (0.56)	<.0001	12 (24.49)	43 (0.94)	<.0001
Assisted Reproduction, n (%)	43 (20.48)	454 (10.24)	<.0001	6 (12.24)	491 (10.69)	0.7254
Family history of PE, n (%)	2 (0.95)	30 (0.68)	0.6546	2 (4.08)	30 (0.65)	0.0442
SLE/APS, n (%)	1 (0.48)	45 (1.01)	0.7224	0 (0.00)	46 (1.00)	1.0000
Smoking, n (%)	1 (0.48)	10 (0.23)	0.3992	0(0.00)	11 (0.24)	1.0000
MAP (mmHg)*	92.89 ± 11.25	82.62 ± 7.29	<.0001	95.71 ± 13.68	82.95 ± 7.61	<.0001
UtA-PI*	1.81 ± 0.59	1.80 ± 0.47	0.6940	1.95 ± 0.61	1.80 ± 0.48	0.0849
PLGF (pg/mL)*	25.75 ± 12.07	33.81 ± 46.25	0.0117	24.87 ± 12.27	33.54 ± 45.51	0.1828
PAPP-A (IU/L)*	3.61 ± 2.37	4.56 ± 2.63	<.0001	3.07 ± 2.64	4.53 ± 2.62	0.0001

*Data are presented as mean ± standard deviation.

### 3.2 Model performance

**Figure 2:**
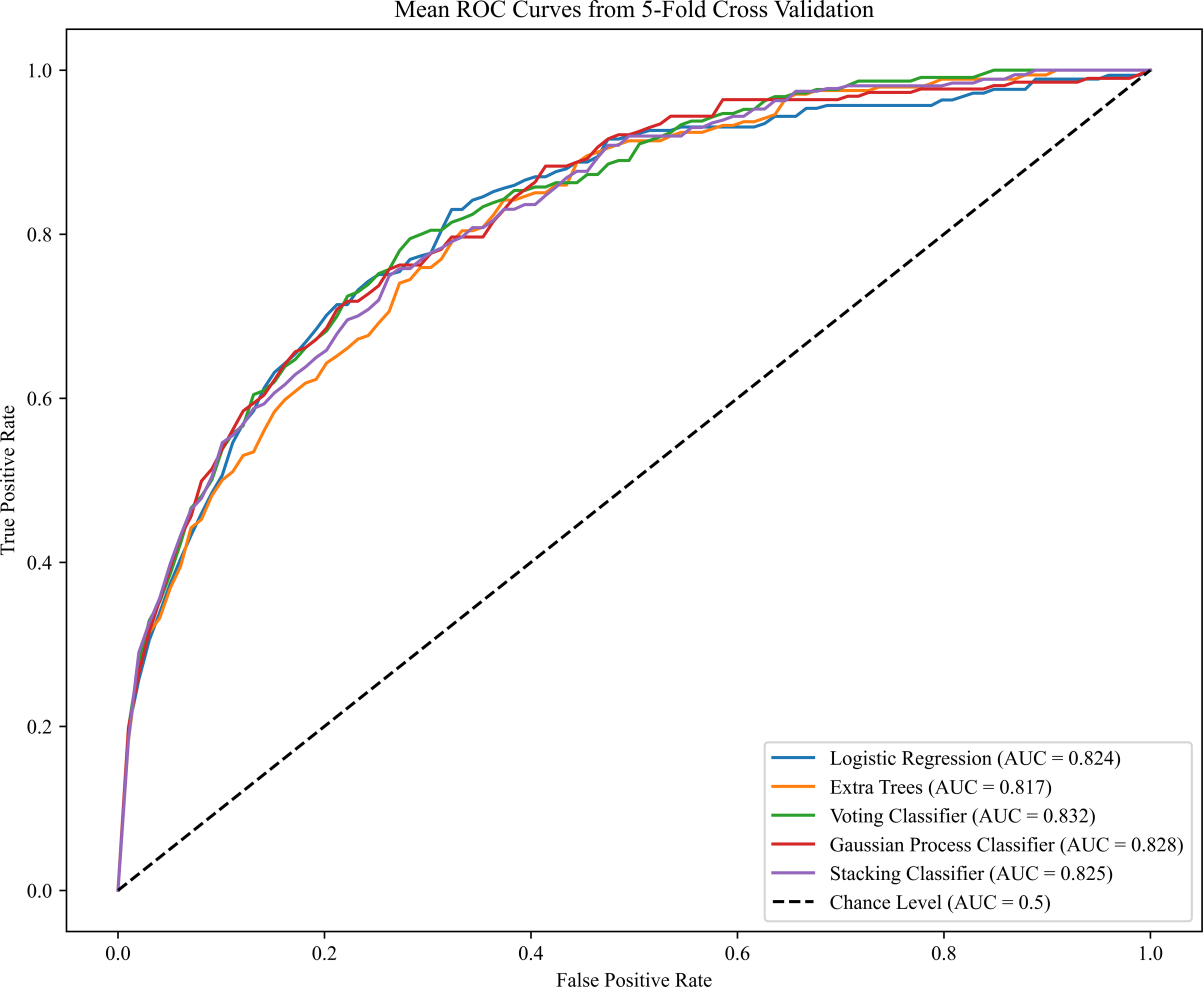
Receiver operating characteristics (ROC) curves for the five ML algorithms in the PE prediction (Maternal Characteristics + MAP + UtA-PI + PLGF + PAPP-A). The plot displays the ROC curve results from the validation set for each of the five model, differentiated by distinct colors. The mean ROC representing the average outcome across all folds within a single epoch of cross-validation. The dashed line is a reference line. AUC is the area under the ROC curve.

The reported area under the AUC, sensitivity, specificity for PE model on the test set are summarized in **Table 2**. The prediction was performed based on the maternal demographic characteristics and medical history data and those characteristics plus MAP, PAPP-A, UtA-PI and PLGF respectively. The AUC increased consistently with the addition of the biomarkers. Specifically, the AUC for PE model in VC algorithms increased from 0.746 to 0.814 when MAP was added in the model, the sensitivity increased from 0.678 to 0.755, but the specificity was remained unchanged at 0.866. When adding the other biomarkers for VC algorithms, the results were slightly improved (AUC of 0.831 and sensitivity of 0.770). Compared to other algorithms, the LR algorithm performed better in terms of AUC in most model scenarios for all PE prediction, especially achieving the best AUC in the scenario of Maternal Characteristics plus MAP (LR: AUC=0.816; ETC: AUC=0.811; VC: AUC=0.814; GPC: AUC=0.814; SC: AUC=0.811).ROC plots with respective AUC of LR, ETC, VC, GPC and SC algorithms for PE model with maternal characteristics plus all biomarkers are shown in **Figure 2**. The AUC value for each of the LR, ETC, VC, GPC and SC algorithms was 0.824, 0.817, 0.832, 0.828 and 0.825 respectively. The performance results of the term PE model are shown in the **Supplementary Table S1**.

**Table 2:** Performance of machine learning algorithms in the preeclampsia model.

PE-All	Algorithm	AUC (95% CI)	Sensitivity (95% CI)	Specificity (95% CI)	10% FPR (95% CI)	20% FPR (95% CI)
Maternal Characteristics	LR	0.749 (0.747, 0.751)	0.659 (0.652, 0.666)	0.750 (0.743, 0.757)	0.400 (0.395, 0.404)	0.544 (0.540, 0.549)
	ETC	0.743 (0.741, 0.745)	0.675 (0.668, 0.682)	0.725 (0.718, 0.731)	0.368 (0.364, 0.372)	0.529 (0.524, 0.533)
	VC	0.746 (0.744, 0.748)	0.678 (0.671, 0.685)	0.722 (0.715, 0.729)	0.374 (0.370, 0.378)	0.529 (0.524, 0.533)
	GPC	0.750 (0.745, 0.754)	0.669 (0.655, 0.684)	0.740 (0.726, 0.754)	0.394 (0.385, 0.403)	0.544 (0.535, 0.553)
	SC	0.746 (0.741, 0.750)	0.668 (0.654, 0.682)	0.733 (0.720, 0.746)	0.376 (0.368, 0.385)	0.532 (0.524, 0.541)
Maternal Characteristics + MAP	LR	0.816 (0.814, 0.818)	0.763 (0.756, 0.769)	0.748 (0.742, 0.754)	0.487 (0.482, 0.491)	0.648 (0.644, 0.652)
	ETC	0.811 (0.810, 0.813)	0.762 (0.755, 0.768)	0.736 (0.729, 0.743)	0.481 (0.477, 0.486)	0.635 (0.631, 0.640)
	VC	0.814 (0.812, 0.816)	0.755 (0.748, 0.761)	0.747 (0.740, 0.753)	0.468 (0.463, 0.472)	0.644 (0.640, 0.649)
	GPC	0.814 (0.811, 0.818)	0.752 (0.740, 0.764)	0.754 (0.742, 0.767)	0.473 (0.464, 0.482)	0.651 (0.643, 0.66)
	SC	0.811 (0.808, 0.815)	0.755 (0.743, 0.767)	0.742 (0.728, 0.755)	0.485 (0.476, 0.493)	0.637 (0.629, 0.645)
Maternal Characteristics + MAP + PAPP-A	LR	0.822 (0.820, 0.824)	0.782 (0.776, 0.787)	0.749 (0.743, 0.754)	0.495 (0.490, 0.499)	0.669 (0.665, 0.673)
	ETC	0.817 (0.815, 0.819)	0.779 (0.773, 0.785)	0.733 (0.727, 0.739)	0.493 (0.489, 0.498)	0.642 (0.638, 0.647)
	VC	0.823 (0.822, 0.825)	0.769 (0.764, 0.775)	0.757 (0.752, 0.762)	0.495 (0.491, 0.499)	0.664 (0.659, 0.668)
	GPC	0.821 (0.817, 0.825)	0.783 (0.772, 0.793)	0.745 (0.734, 0.756)	0.485 (0.475, 0.495)	0.664 (0.656, 0.673)
	SC	0.818 (0.814, 0.822)	0.781 (0.768, 0.794)	0.73 (0.718, 0.743)	0.498 (0.489, 0.507)	0.64 (0.632, 0.649)
Maternal Characteristics + MAP + PAPP-A +UtA-PI	LR	0.826 (0.824, 0.828)	0.776 (0.77, 0.781)	0.763 (0.757, 0.769)	0.506 (0.501, 0.51)	0.68 (0.676, 0.684)
	ETC	0.818 (0.816, 0.82)	0.784 (0.778, 0.789)	0.729 (0.723, 0.736)	0.491 (0.487, 0.496)	0.642 (0.638, 0.647)
	VC	0.826 (0.825, 0.828)	0.763 (0.758, 0.768)	0.766 (0.761, 0.771)	0.508 (0.504, 0.513)	0.671 (0.667, 0.675)
	GPC	0.826 (0.822, 0.829)	0.766 (0.755, 0.778)	0.765 (0.754, 0.776)	0.507 (0.498, 0.516)	0.674 (0.665, 0.683)
	SC	0.822 (0.818, 0.826)	0.779 (0.767, 0.790)	0.741 (0.730, 0.752)	0.502 (0.492, 0.511)	0.654 (0.645, 0.663)
Maternal Characteristics + MAP + PAPP-A +UtA-PI+PLGF	LR	0.826 (0.824, 0.828)	0.777 (0.772, 0.783)	0.763 (0.757, 0.768)	0.506 (0.502, 0.511)	0.681 (0.677, 0.685)
	ETC	0.819 (0.817, 0.821)	0.787 (0.781, 0.793)	0.728 (0.722, 0.734)	0.491 (0.486, 0.496)	0.642 (0.637, 0.646)
	VC	0.831 (0.829, 0.832)	0.77 (0.765, 0.775)	0.769 (0.765, 0.774)	0.513 (0.509, 0.518)	0.681 (0.677, 0.685)
	GPC	0.826 (0.823, 0.83)	0.772 (0.761, 0.783)	0.759 (0.747, 0.771)	0.508 (0.499, 0.518)	0.674 (0.666, 0.682)
	SC	0.823 (0.820, 0.827)	0.772 (0.76, 0.784)	0.752 (0.741, 0.764)	0.503 (0.494, 0.512)	0.655 (0.646, 0.663)

LR, Logistic Regression; ETC, Extra Trees Classifier; VC, Voting Classifier; GPC, Gaussian Process Classifier; SC, Stacking Classifier.

**Figure 3:**
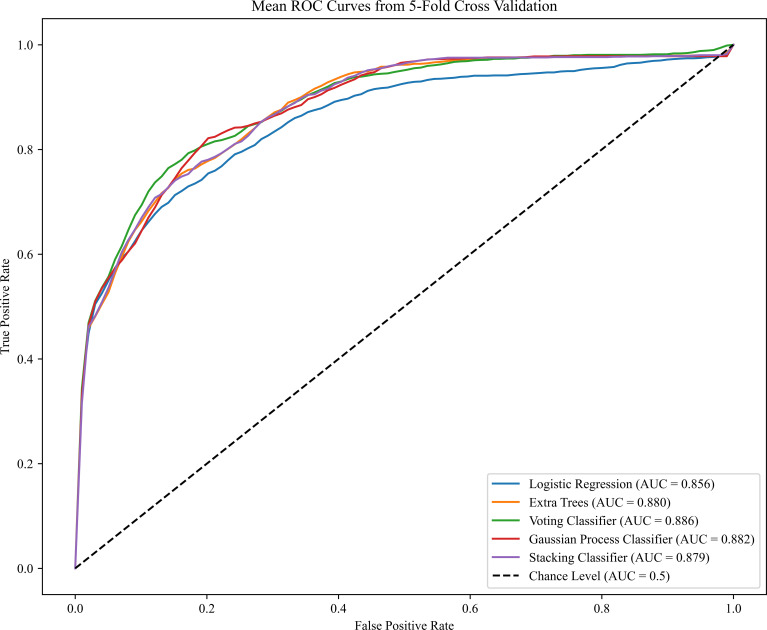
Receiver operating characteristics (ROC) curves for the ML learning algorithms in the preterm PE prediction (Maternal Characteristics + MAP + UtA-PI + PLGF + PAPP-A). The plot displays the ROC curve results from the validation set for each of the five model, differentiated by distinct colors. The mean ROC representing the average outcome across all folds within a single epoch of cross-validation. The dashed line is a reference line. AUC is the area under the ROC curve.

The performance results of the preterm PE model are presented in **Table 3**. Compared to the PE prediction model, the evaluation indicators of preterm PE predictive model in VC algorithms based on the maternal characteristics plus MAP have increased (AUC of 0.842 and sensitivity of 0.762). Notably, the AUC value for predicting PE with maternal characteristics plus the all biomarkers we used was highest in the VC and GPC (**Figure 3**). When the prediction performances were compared among the five algorithms, the VC algorithms had the best performance for predicting preterm PE. The AUC of the VC algorithms was 0.884, sensitivity was 0.860, specificity was 0.834. Details about the model’s calibration are provided in the Appendix, specifically within **Supplementary Tables S2**-**5**.

**Table 3:** Performance of machine learning algorithms in the preterm preeclampsia model.

Preterm PE	Algorithm	AUC (95% CI)	Sensitivity (95% CI)	Specificity (95% CI)	10% FPR (95% CI)	20% FPR (95% CI)
Maternal Characteristics	LR	0.804 (0.798, 0.809)	0.735 (0.726, 0.744)	0.848 (0.84, 0.856)	0.519 (0.51, 0.528)	0.617 (0.608, 0.625)
	ETC	0.799 (0.794, 0.805)	0.719 (0.710, 0.728)	0.864 (0.857, 0.872)	0.543 (0.533, 0.552)	0.647 (0.638, 0.655)
	VC	0.788 (0.783, 0.793)	0.698 (0.689, 0.708)	0.866 (0.858, 0.874)	0.508 (0.499, 0.517)	0.593 (0.585, 0.602)
	GPC	0.802 (0.791, 0.812)	0.714 (0.695, 0.733)	0.862 (0.846, 0.877)	0.515 (0.497, 0.534)	0.605 (0.588, 0.623)
	SC	0.765 (0.754, 0.777)	0.676 (0.657, 0.695)	0.872 (0.855, 0.888)	0.496 (0.478, 0.515)	0.576 (0.559, 0.593)
Maternal Characteristics + MAP	LR	0.836 (0.832, 0.841)	0.779 (0.770, 0.787)	0.852 (0.845, 0.859)	0.571 (0.562, 0.579)	0.664 (0.656, 0.672)
	ETC	0.839 (0.834, 0.843)	0.773 (0.765, 0.781)	0.864 (0.858, 0.87)	0.584 (0.576, 0.593)	0.674 (0.666, 0.681)
	VC	0.842 (0.838, 0.846)	0.762 (0.755, 0.77)	0.866 (0.859, 0.872)	0.577 (0.568, 0.585)	0.655 (0.647, 0.663)
	GPC	0.842 (0.833, 0.851)	0.782 (0.765, 0.800)	0.849 (0.839, 0.860)	0.565 (0.548, 0.581)	0.688 (0.672, 0.703)
	SC	0.841 (0.834, 0.849)	0.777 (0.762, 0.792)	0.86 (0.848, 0.871)	0.575 (0.559, 0.591)	0.67 (0.657, 0.683)
Maternal Characteristics + MAP + PAPP-A	LR	0.853 (0.849, 0.857)	0.828 (0.821, 0.836)	0.829 (0.822, 0.835)	0.577 (0.568, 0.585)	0.695 (0.688, 0.703)
	ETC	0.87 (0.866, 0.873)	0.853 (0.846, 0.86)	0.818 (0.811, 0.824)	0.589 (0.581, 0.598)	0.715 (0.708, 0.722)
	VC	0.877 (0.874, 0.881)	0.852 (0.845, 0.859)	0.828 (0.821, 0.835)	0.608 (0.600, 0.617)	0.715 (0.708, 0.722)
	GPC	0.87 (0.863, 0.877)	0.847 (0.834, 0.86)	0.828 (0.818, 0.838)	0.585 (0.569, 0.601)	0.727 (0.714, 0.740)
	SC	0.87 (0.862, 0.877)	0.842 (0.827, 0.857)	0.826 (0.812, 0.839)	0.585 (0.567, 0.603)	0.704 (0.689, 0.718)
Maternal Characteristics + MAP + PAPP-A +UtA-PI	LR	0.86 (0.856, 0.864)	0.833 (0.826, 0.841)	0.826 (0.819, 0.832)	0.589 (0.58, 0.597)	0.702 (0.695, 0.71)
	ETC	0.878 (0.874, 0.881)	0.863 (0.856, 0.870)	0.816 (0.809, 0.823)	0.603 (0.595, 0.612)	0.715 (0.708, 0.721)
	VC	0.889 (0.885, 0.892)	0.868 (0.861, 0.874)	0.834 (0.828, 0.84)	0.623 (0.615, 0.632)	0.741 (0.735, 0.748)
	GPC	0.883 (0.877, 0.890)	0.874 (0.862, 0.886)	0.821 (0.809, 0.833)	0.602 (0.586, 0.619)	0.739 (0.725, 0.753)
	SC	0.876 (0.87, 0.883)	0.864 (0.85, 0.877)	0.814 (0.8, 0.828)	0.589 (0.573, 0.605)	0.714 (0.7, 0.728)
Maternal Characteristics + MAP + PAPP-A +UtA-PI+PLGF	LR	0.858 (0.854, 0.862)	0.828 (0.821, 0.836)	0.829 (0.823, 0.836)	0.583 (0.575, 0.591)	0.698 (0.691, 0.706)
	ETC	0.878 (0.875, 0.881)	0.868 (0.862, 0.875)	0.814 (0.807, 0.821)	0.603 (0.595, 0.611)	0.717 (0.709, 0.724)
	VC	0.884 (0.881, 0.888)	0.860 (0.853, 0.866)	0.834 (0.828, 0.841)	0.625 (0.617, 0.632)	0.729 (0.723, 0.736)
	GPC	0.883 (0.876, 0.889)	0.870 (0.858, 0.882)	0.822 (0.81, 0.834)	0.599 (0.582, 0.616)	0.743 (0.730, 0.757)
	SC	0.878 (0.871, 0.885)	0.867 (0.853, 0.881)	0.813 (0.799, 0.828)	0.595 (0.578, 0.612)	0.718 (0.704, 0.733)

LR, Logistic Regression; ETC, Extra Trees Classifier; VC, Voting Classifier; GPC, Gaussian Process Classifier; SC, Stacking Classifier.

The FMF competing risk models for predicting PE and preterm PE, incorporating maternal characteristics plus all biomarkers, exhibit AUCs of 0.797 and 0.856, respectively. The performance for PE or preterm PE using ML algorithms in this study was similar to that achieved by the FMF competing risk model under the same predictive factors. Details about the performances of FMF competing risk model for PE and preterm PE prediction are provided in **Supplementary Table S6**.

### 3.3 Contribution of variables to prediction accuracy

**Figure 4:**
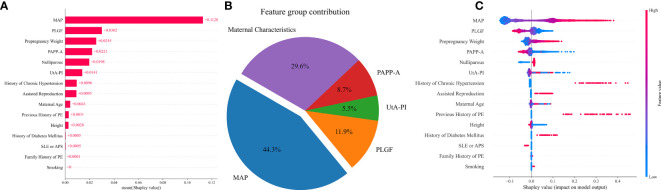
Contribution of variables to PE prediction based on Shapley values. **(A)** The mean Shapley values of predictors of PE based on the VC model. Input variables are ordered by their mean Shapley value, which reflect the relative importance of the predictors in PE predictive model. **(B)** The pie chart of the feature group contribution based on the VC model for PE prediction. the average feature group contribution was calculated based on the mean absolute Shapley values for each feature group. **(C)** Shapley summary plot for PE predictors based on the VC model. The input predictors (Y-axis) are ordered by the mean absolute Shapley values, which represent their average influence on the model output. Values of the predictor for each subject are colored by their Shapley values, red indicating high contribution and blue indicating low contribution. Positive Shapley values indicate increased risk for PE and negative Shapley values indicate decreased risk for PE.

The influence of each variable on prediction accuracy was evaluated by Shapley values, a large absolute Shapley value indicates that the input variable typically contributes more to decision making. The Shapley values represent the average contribution to the score of each input variable when computed with different combinations of the other variables. Contributions of variables to PE prediction are shown in **Figure 4**. The highest contribution was provided by MAP, for which a high value led to a high risk for preterm PE, followed in order by PLGF, pre-pregnancy weight, PAPP-A, nulliparous, UtA-PI, history of chronic hypertension, assisted reproduction, maternal age, and previous history of PE (**Figures 4A, C**). From the feature contribution distribution chart (**Figure 4B**), the contribution ratio of MAP for PE prediction is 44.3%, with the maternal characteristics contributing 29.6%. This is followed by three biomarkers: PLGF, PAPP-A, and UtA-PI, which account for 11.9%, 8.7%, and 5.5%, respectively.

**Figure 5:**
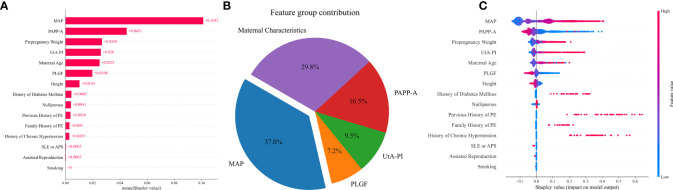
Contribution of variables to preterm PE prediction based on Shapley values. **(A)** The mean Shapley values of predictors of preterm PE based on the VC model. Input variables are ordered by their mean Shapley value, which reflect the relative importance of the predictors in preterm PE predictive model. **(B)** The pie chart of the feature group contribution based on the VC model for preterm PE prediction. the average feature group contribution was calculated based on the mean absolute Shapley values for each feature group. **(C)** Shapley summary plot for preterm PE predictors based on the VC model. The input predictors (Y-axis) are ordered by the mean absolute Shapley values, which represent their average influence on the model output. Values of the predictor for each subject are colored by their Shapley values, red indicating high contribution and blue indicating low contribution. Positive Shapley values indicate increased risk for preterm PE and negative Shapley values indicate decreased risk for preterm PE.

The relative importance of the selected variables in the preterm PE prediction model is described in **Figure 5**. The most important predictive factor is still MAP, followed by PAPP-A, pre-pregnancy weight, UtA-PI, maternal age, and PLGF. Other indicators, such as smoking, had a very limited contribution (**Figures 5A, C**). The contributions of MAP and maternal characteristics to the prediction of preterm PE are 37.0% and 29.8% respectively. Among the three biomarkers, PAPP-A has the highest proportion at 16.5%, while the remaining PLGF and UtA-PI account for 7.2% and 9.5% respectively (**Figure 5B**).

### 3.4 Comparison to previous studies

We searched PubMed for articles on prediction models for PE published between 2019 and 2023, including both developed and validated models. We retrieved a total of 242 publications within the past five years from the PubMed database using the search terms (Preeclampsia [Title]) AND (prediction [Title]). Ultimately, nine relevant publications were selected based on the inclusion and exclusion criteria (**Table 4**). In the articles predicting all PE, we identified that the Ansbacher-Feldman (19) study from the UK and the Gil, M. M (20) study from Spain employed the same clinical biomarkers as we did. The predictive model AUCs in these two studies were 0.817 and 0.848, respectively, and the detection rates at a 10% false positive rate were 0.529 and 0.548, respectively, which are comparable to the levels of VC algorithm in our study (AUC=0.831, DR at 10%FPR=0.513). Similarly, the two aforementioned studies used the same predictive factors to forecast pre-term PE, with AUCs of 0.909 and 0.912, respectively. The detection rates at a 10% false positive rate were 0.753 and 0.778, slightly higher than VC algorithm in our study (AUC=0.884, DR at 10%FPR=0.625).

**Table 4:** Predictive performances of the Voting Classifier model for the PE compared to those from previous studies.

Source	Data Source	Algorithm	SampleSize	ROC (95% CI)	DR at 10%FPR	Predictive factors
All PE						
Adi L. TARCAet al,2022 (36)	USA	Multivariable Poisson regression	1150	0.76(0.71–0.81)	0.44 (0.35–0.54)	MC+MAP+PLGF+sVEGFR-1+sEng
Ansbacher-Feldman, Z.et al,2022 (19)	UK	Artificial neural network	60789	0.817 (0.797–0.837)	0.529 (0.482–0.576)	MC+MAP+PLGF+PAPP-A+UtA-PI
Piya Chaemsaithong,et al,2019 (32)	Asian	FMF Bayes theorem-based model	10935	0.769(0.761–0.777)	0.493(0.429–0.559)	MC+MAP+PLGF+UtA-PI
Liu,M,et al,2022 (23)	CHINA	Random Forest	11152	0.86 (0.80–0.82)	NA	MC+MAP+β-HCG+PAPP-A++UtA-PI
Melinte-Popescu, A. S,et al,2023 (21)	Romania	support vector machine (SVM)	233	0.98	NA	MC+MAP+PAPP-A+UtA-PI+PLGF+PP-13
Torres-Torres, J,et al,2023 (22)	Mexico	elastic net	3050	0.776(0.724–0.829)	0.481(0.364–0.587)	MC+MAP+UtA-PI+PLGF
Gil, M. M.et al,2023 (20)	Spain	fully connected neural network	10110	0.848(0.822–0.873)	0.548(0.481–0.613)	MC+MAP+UtA-PI+PLGF+PAPP-A
Our Study	CHINA	Voting Classifier	4644	0.831(0.773–0.883)	0.513(0.381–0.643)	MC+MAP+UtA-PI+PLGF+PAPP-A
Our Study	CHINA	Logistic Regression	4644	0.826(0.824–0.828)	0.506(0.502–0.511)	MC+MAP+UtA-PI+PLGF+PAPP-A
Our Study	CHINA	Extra Trees Classifier	4644	0.819(0.817–0.821)	0.491(0.486–0.496)	MC+MAP+UtA-PI+PLGF+PAPP-A
Our Study	CHINA	Gaussian Process Classifier	4644	0.826(0.823–0.830)	0.508(0.499–0.518)	MC+MAP+UtA-PI+PLGF+PAPP-A
Our Study	CHINA	Stacking Classifier	4644	0.823(0.820–0.827)	0.503(0.494–0.512)	MC+MAP+UtA-PI+PLGF+PAPP-A
Pre-term PE						
Adi L. TARCAet al,2022 (36)	USA	Multivariable Poisson regression	1150	0.78(0.70–0.86)	0.55 (0.39–0.70)	MC+MAP+PLGF+sVEGFR-1+sEng
Ansbacher-Feldman, Z.et al,2022 (19)	UK	Artificial neural network	59551	0.909(0.895–0.923)	0.753(0.689–0.817)	MC+MAP+PLGF+PAPP-A+UtA-PI
Jing Zhang.et al,2019 (37)	CHINA	PREDICTOR algorithm	3270	0.901 (0.890–0.911)	0.875(0.474–0.997)	MC+MAP+PLGF+PAPP-A
Piya Chaemsaithong,et al,2019 (32)	Asian	FMF Bayes theorem-based model	10935	0.857 (0.851–0.864)	0.640(0.534–0.747)	MC+MAP+PLGF+UtA-PI
Rezende, K. B. C,et al,2019 (38)	Brazilian	competitive risk model	1531	0.77(0.68–0.86)	NA	MC+MAP+UtA-PI+Crown-rump length
Torres-Torres, J,et al,2023 (22)	Mexico	elastic net	3050	0.883(0.853–0.942)	0.733(0.600–0.837)	MC+MAP+UtA-PI+PLGF
Gil, M. M.et al,2023 (20)	Spain	fully connected neural network	10110	0.912(0.880–0.944)	0.778(0.664–0.867)	MC+MAP+UtA-PI+PLGF+PAPP-A
Our Study	CHINA	Logistic Regression	4644	0.858(0.854–0.862)	0.583(0.575–0.591)	MC+MAP+UtA-PI+PLGF+PAPP-A
Our Study	CHINA	Extra Trees Classifier	4644	0.878(0.875–0.881)	0.603(0.595–0.611)	MC+MAP+UtA-PI+PLGF+PAPP-A
Our Study	CHINA	Voting Classifier	4644	0.884(0.881–0.888)	0.625(0.617–0.632)	MC+MAP+UtA-PI+PLGF+PAPP-A
Our Study	CHINA	Gaussian Process Classifier	4644	0.883(0.876–0.889)	0.599(0.582–0.616)	MC+MAP+UtA-PI+PLGF+PAPP-A
Our Study	CHINA	Stacking Classifier	4644	0.878(0.871–0.885)	0.595(0.578–0.612)	MC+MAP+UtA-PI+PLGF+PAPP-A

^a^DR, Detection rate; FPR, False-positive rate; MC, Maternal Characteristic; MAP, mean arterial pressure; PAPP-A, pregnancy-associated plasma protein-A; PLGF, placental growth factor; UtA-PI, uterine artery pulsatility index; β-HCG, β-human chorionic gonadotropin; sVEGFR, soluble vascular endothelial growth factor receptor; sEng, soluble endoglin.

## 4 Discussion

In this study, we successfully developed a fully automated prediction model for all PE and preterm PE by using various ML algorithms. Compared with other prediction algorithms, the AUC and detection rate at 10% FPR of the VC algorithm showed better performance in the prediction of PE (AUC=0.831, DR at 10%FPR=0.513) and preterm PE (AUC=0.884, DR at 10%FPR=0.625). For predicting PE and preterm PE, the most crucial predictive factors were MAP and maternal characteristics. In predicting all PE, the contribution of PLGF was higher than PAPP-A (11.9% versus 8.7%), whereas the situation was reversed in the prediction of preterm PE (7.2% versus 16.5%).

The guidelines of both the International Society for the Study of Hypertension in Pregnancy (39) and the International Federation of Gynecology and Obstetrics (FIGO) (6) emphasized the critical importance of early prediction and prevention of PE for reducing the incidence of PE, and directly improving the health outcomes of the maternal and newborns. FMF competing risk model had provided significant insights and an effective method for PE prediction (40). Our study showed that the AUC for the PE prediction model, based on the competing risk model with maternal characteristics plus all biomarkers, was 0.797, while the AUC for the preterm PE prediction model was 0.856. Some researchers had adopted other predictive algorithms for PE prediction, demonstrating similar model performance. For example, Tarca, A. L. et al. achieved good performance in predicting PE using a multivariate Poisson regression model based on maternal baseline, biophysical and biochemical biomarkers (36). Our study constructed prediction models for PE using several ML algorithms and obtained similar model performance. However, the biomarkers values in the ML algorithms do not need to be expressed as multiples of the median (MoM) and adjusted for gestational age and various maternal factors, the application scenarios for ML algorithms may be more extensive. ML algorithms can particularly utilize raw clinical data directly and are capable of analyzing complex, nonlinear, and high-dimensional data, aligning with the practical scenarios of PE clinical predictions. Additionally, ML algorithms learn and adapt to new data, leading to continuous improvement in predictions over time. Therefore, establishing rapid and simple prediction tools, such as online web pages, based on ML algorithms is suitable for carrying out large-scale screening and prediction of PE.

Apart from the prediction model algorithms, the predictive factors included are also crucial for predicting PE. The models that are overly complex or costly are not practical for screening large populations at the community level. A required predictive model should be both cost-effective and highly sensitive, meaning that the predictors within the model should be low-cost and yet acceptable in terms of accuracy (41). Our research findings indicate that in the prediction model for PE, the combination of maternal characteristics, MAP, PAPP-A as predictors can achieve an AUC of 0.82. For preterm PE, this combination can achieve an AUC of 0.87. The increase in predictive effect was limited by adding the other parameters. PLGF and UtA-PI could only increase the AUC to 0.83 for PE prediction and to 0.88 for preterm PE prediction. J. Torres-Torres’s study (22) also found that in PE prediction, the AUC was 0.786 when the predictive factors included maternal characteristics and MAP. However, when PLGF and UtA-PI were added, the AUC decreased to 0.778. In Benkő, Z’s study (42) on the prediction of preterm PE in twin pregnancy, a similar finding was observed. The AUC was 0.742 when the prediction was based on maternal characteristics and MAP alone. However, with the inclusion of PLGF, UtA-PI, and PAPP-A, the AUC only increased to 0.776. In summary, based on the performance of the predictive model, the combination of maternal baseline, MAP, and PAPP-A meets the expectations for predicting PE and preterm PE. Considering the interpretability and parsimony of the model, as well as the real-world requirements for low-cost effectiveness analysis in clinical applications, we recommend the combination of maternal baseline characteristics with MAP and PAPP-A as predictive factors for the prediction of PE and preterm PE.

The strengths of this study are listed as follows: Firstly, the study was designed as a prospective cohort study. Strict quality control was maintained in data collection throughout the research process to ensure that the data used for modeling were objective and credible; Secondly, the model underwent rigorous validation using a 5-fold cross-validation with an 8:2 train-test split. This stringent testing ensures that the model is not only accurate but robust, instilling confidence in its practical application; Finally, despite the often “black box” nature of ML models, we employed SHAP values to enhance the interpretability of our model. This transparency is crucial for clinical adoption, offering insights into the underlying factors driving the predictions. The main limitation of this study is the relatively small sample size from a single center, lacking external data validation. It is essential to test the model on a more varied dataset to confirm its applicability across different populations. It is anticipated that it will be necessary to assess the model performance in diverse real-world settings to confirm its efficacy and reliability.

## 5 Conclusions

In conclusion, our study offers several automated machine-learning algorithms to make PE and preterm PE predictions more accessible, cost-effective, and reliable in the first-trimester. The integration of maternal baseline, MAP, and PAPP-A into the predictive model could potentially revolutionize PE screening, making it more accessible and reliable, especially in developing countries where resources and specialized training are limited.

## Data availability statement

The datasets used during the current study are available from the corresponding author on reasonable request.

## Ethics statement

The studies involving humans were approved by the institutional review board of Nanjing Drum Tower Hospital. The studies were conducted in accordance with the local legislation and institutional requirements. Written informed consent for participation in this study was provided by the participants’ legal guardians/next of kin.

## Author contributions

TL: Formal analysis, Software, Writing – original draft. MX: Formal analysis, Methodology, Validation, Writing – review & editing. YuW: Data curation, Project administration, Writing – review & editing. YaW: Data curation, Project administration, Writing – review & editing. HT: Data curation, Project administration, Writing – review & editing. HD: Data curation, Project administration, Writing – review & editing. GZ: Conceptualization, Funding acquisition, Writing – review & editing. MZ: Conceptualization, Supervision, Writing – review & editing. YH: Conceptualization, Supervision, Writing – review & editing.
