# Supplementary material for: Prediction model of preeclampsia using machine learning based methods: a population based cohort study in China
Source: Front Endocrinol (Lausanne). 2024 Jun 11;15:1345573. doi: 10.3389/fendo.2024.1345573 (PMC11198873; doi:10.3389/fendo.2024.1345573)
Supplement: Supplementary file 1 [file Table_1.docx]

**Supplemental Table 1 Performance of machine learning algorithms in the term preeclampsia model**

| **Term PE** | **Algorithm** | **AUC**  **(95% CI)** | **Sensitivity**  **(95% CI)** | **Specificity**  **(95% CI)** | **10% FPR**  **(95% CI)** | **20% FPR**  **(95% CI)** |
| --- | --- | --- | --- | --- | --- | --- |
| Maternal Characteristics | LR | 0.731  ( 0.728 , 0.733 ) | 0.664  ( 0.656 , 0.672 ) | 0.726  ( 0.718 , 0.734 ) | 0.345  ( 0.340, 0.350 ) | 0.507  ( 0.502 , 0.512 ) |
|  | ETC | 0.725  ( 0.722 , 0.728 ) | 0.699  ( 0.691 , 0.707 ) | 0.684  ( 0.676 , 0.691 ) | 0.331  ( 0.326 , 0.336 ) | 0.482  ( 0.478 , 0.487 ) |
|  | VC | 0.729  ( 0.726 , 0.732 ) | 0.705  ( 0.697 , 0.714 ) | 0.678  ( 0.669 , 0.687 ) | 0.337  ( 0.332 , 0.342 ) | 0.486  ( 0.481 , 0.491 ) |
|  | GPC | 0.731  ( 0.726 , 0.737 ) | 0.683  ( 0.666 , 0.699 ) | 0.708  ( 0.692 , 0.723 ) | 0.345  ( 0.336 , 0.354 ) | 0.508  ( 0.499 , 0.518 ) |
|  | SC | 0.726  ( 0.721 , 0.731 ) | 0.697  ( 0.682 , 0.711 ) | 0.689  ( 0.675 , 0.704 ) | 0.333  ( 0.324 , 0.342 ) | 0.487  ( 0.476 , 0.497 ) |
| Maternal Characteristics + MAP | LR | 0.805  ( 0.802 , 0.807 ) | 0.781  ( 0.774 , 0.787 ) | 0.719  ( 0.712 , 0.726 ) | 0.443  ( 0.438 , 0.448 ) | 0.620  ( 0.615 , 0.625 ) |
|  | ETC | 0.796  ( 0.794 , 0.798 ) | 0.805  ( 0.798 , 0.811 ) | 0.678  ( 0.672 , 0.685 ) | 0.423  ( 0.418 , 0.428 ) | 0.589  ( 0.584 , 0.594 ) |
|  | VC | 0.801  ( 0.799 , 0.803 ) | 0.77  ( 0.763 , 0.778 ) | 0.71  ( 0.702 , 0.718 ) | 0.422  ( 0.417 , 0.427 ) | 0.605  ( 0.600 , 0.609 ) |
|  | GPC | 0.800  ( 0.797 , 0.804 ) | 0.784  ( 0.770 , 0.798 ) | 0.705  ( 0.691 , 0.718 ) | 0.431  ( 0.422 , 0.441 ) | 0.615  ( 0.607 , 0.624 ) |
|  | SC | 0.797  ( 0.793 , 0.801 ) | 0.787  ( 0.772 , 0.802 ) | 0.697  ( 0.682 , 0.712 ) | 0.439  ( 0.429 , 0.449 ) | 0.594  ( 0.585 , 0.603 ) |
| Maternal Characteristics + MAP + PAPP-A | LR | 0.808  ( 0.806 , 0.81 ) | 0.79  ( 0.784 , 0.796 ) | 0.724  ( 0.718 , 0.731 ) | 0.45  ( 0.445 , 0.455 ) | 0.631  ( 0.626 , 0.635 ) |
|  | ETC | 0.798  ( 0.795 , 0.800) | 0.809  ( 0.803 , 0.814 ) | 0.686  ( 0.68 , 0.692 ) | 0.422  ( 0.417 , 0.428 ) | 0.587  ( 0.582 , 0.592 ) |
|  | VC | 0.804  ( 0.802 , 0.806 ) | 0.773  ( 0.768 , 0.779 ) | 0.724  ( 0.718 , 0.73 ) | 0.434  ( 0.429 , 0.439 ) | 0.613  ( 0.609 , 0.618 ) |
|  | GPC | 0.803  ( 0.799 , 0.807 ) | 0.785  ( 0.773 , 0.797 ) | 0.718  ( 0.707 , 0.730 ) | 0.436  ( 0.426 , 0.446 ) | 0.618  ( 0.61 , 0.627 ) |
|  | SC | 0.799  ( 0.795 , 0.803 ) | 0.801  ( 0.788 , 0.813 ) | 0.696  ( 0.684 , 0.708 ) | 0.436  ( 0.427 , 0.446 ) | 0.594  ( 0.585 , 0.604 ) |
| Maternal Characteristics + MAP + PAPP-A  +UtA-PI | LR | 0.81  ( 0.808 , 0.812 ) | 0.783  ( 0.777 , 0.789 ) | 0.736  ( 0.73 , 0.742 ) | 0.457  ( 0.452 , 0.461 ) | 0.644  ( 0.639 , 0.648 ) |
|  | ETC | 0.797  ( 0.795 , 0.800 ) | 0.807  ( 0.801 , 0.813 ) | 0.686  ( 0.68 , 0.692 ) | 0.42  ( 0.414 , 0.425 ) | 0.587  ( 0.582 , 0.592 ) |
|  | VC | 0.805  ( 0.803 , 0.807 ) | 0.767  ( 0.761 , 0.773 ) | 0.731  ( 0.725 , 0.737 ) | 0.441  ( 0.436 , 0.446 ) | 0.614  ( 0.609 , 0.619 ) |
|  | GPC | 0.806  ( 0.802 , 0.810 ) | 0.772  ( 0.76 , 0.784 ) | 0.737  ( 0.725 , 0.749 ) | 0.45  ( 0.441 , 0.46 ) | 0.631  ( 0.621 , 0.640 ) |
|  | SC | 0.801  ( 0.797 , 0.806 ) | 0.795  ( 0.781 , 0.808 ) | 0.705  ( 0.693 , 0.718 ) | 0.448  ( 0.437 , 0.458 ) | 0.600  ( 0.589 , 0.610) |
| Maternal Characteristics + MAP + PAPP-A  +UtA-PI+PLGF | LR | 0.81  ( 0.808 , 0.812 ) | 0.784  ( 0.777 , 0.79 ) | 0.736  ( 0.729 , 0.742 ) | 0.455  ( 0.450 , 0.460 ) | 0.646  ( 0.642 , 0.651 ) |
|  | ETC | 0.799  ( 0.796 , 0.801 ) | 0.81  ( 0.804 , 0.815 ) | 0.686  ( 0.681 , 0.692 ) | 0.419  ( 0.414 , 0.424 ) | 0.587  ( 0.582 , 0.592 ) |
|  | VC | 0.81  ( 0.808 , 0.812 ) | 0.77  ( 0.764 , 0.776 ) | 0.736  ( 0.730 , 0.741 ) | 0.447  ( 0.442 , 0.452 ) | 0.621  ( 0.616 , 0.626 ) |
|  | GPC | 0.807  ( 0.802 , 0.812 ) | 0.783  ( 0.771 , 0.795 ) | 0.728  ( 0.716 , 0.741 ) | 0.449  ( 0.438 , 0.460 ) | 0.632  ( 0.622 , 0.643 ) |
|  | SC | 0.802  ( 0.798 , 0.807 ) | 0.804  ( 0.792 , 0.816 ) | 0.697  ( 0.685 , 0.708 ) | 0.446  ( 0.436 , 0.457 ) | 0.599  ( 0.588 , 0.609 ) |

***Abbreviation:*** LR, Logistic Regression; ETC, Extra Trees Classifier; VC, Voting Classifier; GPC, Gaussian Process Classifier; SC, Stacking Classifier.
