# Supplementary material for: Prediction model of preeclampsia using machine learning based methods: a population based cohort study in China
Source: Front Endocrinol (Lausanne). 2024 Jun 11;15:1345573. doi: 10.3389/fendo.2024.1345573 (PMC11198873; doi:10.3389/fendo.2024.1345573)
Supplement: Supplementary file 2 [file Table_2.docx]

**Supplemental Table 2 Calibration Performance of machine learning algorithms in the all PE predictive model**

| **All PE** | **Algorithm** | **Calibration** | | |
| --- | --- | --- | --- | --- |
|  |  | **Brier score**  **(95% CI)** | **Slope**  **(95% CI)** | **Intercept**  **(95% CI)** |
| Maternal Characteristics + MAP + UtA-PI + PLGF + PAPP-A | Logistic Regression | 0.038  [0.037-0.038] | 0.800  [0.786-0.813] | 0.034  [0.032-0.035] |
|  | Extra Trees Classifier | 0.038  [0.037-0.038] | 0.823  [0.808-0.836] | 0.025  [0.022-0.026] |
|  | Voting Classifier | 0.038  [0.038-0.038] | 0.861  [0.842-0.880] | 0.006  [0.004-0.009] |
|  | Gaussian Process Classifier | 0.039  [0.038-0.040] | 0.614  [0.563-0.665] | 0.084  [0.076-0.092] |
|  | Stacking Classifier | 0.039  [0.038-0.040] | 0.622  [0.558-0.685] | 0.078  [0.069-0.086] |

Brier Score is a measure of the accuracy of probabilistic predictions. The Brier Score ranges from 0 for a perfect model to 1 for the worst model. Lower scores are better; These refer to the calibration slope and intercept when fitting a linear calibration curve to predicted probabilities against observed outcomes. If you were to fit a linear regression to this plot, the slope should ideally be 1, and the intercept should be 0 for a perfectly calibrated model.
