# Supplementary material for: Prediction model of preeclampsia using machine learning based methods: a population based cohort study in China
Source: Front Endocrinol (Lausanne). 2024 Jun 11;15:1345573. doi: 10.3389/fendo.2024.1345573 (PMC11198873; doi:10.3389/fendo.2024.1345573)
Supplement: Supplementary file 3 [file Table_3.docx]

**Supplemental Table 3 Mean model calibration by fold in all PE prediction model**

| **(PE-All)** | **Mean predicted value** | **Fraction of positives for fold** | | | | | |
| --- | --- | --- | --- | --- | --- | --- | --- |
| Logistic Regression |  | 1 | 2 | 3 | 4 | 5 | Over All |
|  | 0.05 | 0.008 | 0.033 | 0.033 | 0.033 | 0.033 | 0.033 |
|  | 0.15 | 0.188 | 0.108 | 0.108 | 0.108 | 0.108 | 0.108 |
|  | 0.25 | 0.307 | 0.228 | 0.230 | 0.228 | 0.230 | 0.229 |
|  | 0.35 | 0.355 | 0.349 | 0.356 | 0.352 | 0.353 | 0.356 |
|  | 0.45 | 0.263 | 0.436 | 0.440 | 0.435 | 0.430 | 0.429 |
|  | 0.55 | 0.142 | 0.503 | 0.492 | 0.487 | 0.496 | 0.489 |
|  | 0.65 | 0.206 | 0.544 | 0.546 | 0.538 | 0.549 | 0.539 |
|  | 0.75 | 0.448 | 0.596 | 0.587 | 0.590 | 0.595 | 0.592 |
|  | 0.85 | 0.567 | 0.590 | 0.577 | 0.601 | 0.583 | 0.597 |
|  | 0.95 | 0.644 | 0.666 | 0.670 | 0.676 | 0.650 | 0.673 |
| Extra Trees Classifier |  | 1 | 2 | 3 | 4 | 5 | Over All |
|  | 0.05 | 0.007 | 0.032 | 0.032 | 0.032 | 0.032 | 0.032 |
|  | 0.15 | 0.213 | 0.099 | 0.098 | 0.096 | 0.100 | 0.100 |
|  | 0.25 | 0.285 | 0.246 | 0.257 | 0.229 | 0.236 | 0.249 |
|  | 0.35 | 0.244 | 0.467 | 0.520 | 0.381 | 0.458 | 0.444 |
|  | 0.45 | 0.269 | 0.298 | 0.304 | 0.311 | 0.340 | 0.323 |
|  | 0.55 | 0.527 | 0.436 | 0.395 | 0.428 | 0.441 | 0.446 |
|  | 0.65 | 0.545 | 0.572 | 0.597 | 0.569 | 0.553 | 0.559 |
|  | 0.75 | 0.623 | 0.590 | 0.635 | 0.636 | 0.554 | 0.553 |
|  | 0.85 | 0.701 | 0.350 | 0.333 | 0.722 | 0.633 | 0.574 |
|  | 0.95 | 0.779 | 0.662 | 0.672 | 0.804 | 0.768 | 0.743 |
| Voting Classifier |  | 1 | 2 | 3 | 4 | 5 | Over All |
|  | 0.05 | 0.007 | 0.029 | 0.029 | 0.029 | 0.029 | 0.029 |
|  | 0.15 | 0.065 | 0.098 | 0.096 | 0.097 | 0.098 | 0.095 |
|  | 0.25 | 0.285 | 0.178 | 0.184 | 0.182 | 0.197 | 0.191 |
|  | 0.35 | 0.258 | 0.229 | 0.256 | 0.243 | 0.259 | 0.274 |
|  | 0.45 | 0.281 | 0.558 | 0.384 | 0.408 | 0.408 | 0.369 |
|  | 0.55 | 0.514 | 0.625 | 0.425 | 0.606 | 0.476 | 0.571 |
|  | 0.65 | 0.580 | 0.569 | 0.593 | 0.574 | 0.513 | 0.593 |
|  | 0.75 | 0.671 | 0.569 | 0.685 | 0.575 | 0.491 | 0.564 |
|  | 0.85 | 0.761 | 0.750 | 0.667 | 0.500 | 0.502 | 0.496 |
|  | 0.95 | 0.852 | 0.895 | 0.845 | 0.778 | 0.691 | 0.761 |
| Gaussian Process Classifier |  | 1 | 2 | 3 | 4 | 5 | Over All |
|  | 0.05 | 0.009 | 0.035 | 0.035 | 0.035 | 0.034 | 0.035 |
|  | 0.15 | 0.279 | 0.143 | 0.156 | 0.148 | 0.141 | 0.152 |
|  | 0.25 | 0.298 | 0.268 | 0.222 | 0.325 | 0.262 | 0.250 |
|  | 0.35 | 0.260 | 0.404 | 0.283 | 0.359 | 0.426 | 0.348 |
|  | 0.45 | 0.242 | 0.499 | 0.496 | 0.372 | 0.343 | 0.428 |
|  | 0.55 | 0.146 | 0.307 | 0.509 | 0.364 | 0.420 | 0.403 |
|  | 0.65 | 0.208 | 0.501 | 0.472 | 0.667 | 0.449 | 0.517 |
|  | 0.75 | 0.196 | 0.420 | 0.767 | 0.731 | 0.533 | 0.593 |
|  | 0.85 | 0.308 | 0.581 | 0.600 | 0.550 | 0.710 | 0.656 |
|  | 0.95 | 0.559 | 0.573 | 0.530 | 0.460 | 0.613 | 0.593 |
| Stacking Classifier |  | 1 | 2 | 3 | 4 | 5 | Over All |
|  | 0.05 | 0.008 | 0.035 | 0.034 | 0.034 | 0.034 | 0.035 |
|  | 0.15 | 0.312 | 0.157 | 0.153 | 0.149 | 0.136 | 0.143 |
|  | 0.25 | 0.252 | 0.206 | 0.294 | 0.307 | 0.257 | 0.286 |
|  | 0.35 | 0.234 | 0.179 | 0.136 | 0.355 | 0.239 | 0.302 |
|  | 0.45 | 0.260 | 0.390 | 0.217 | 0.396 | 0.283 | 0.247 |
|  | 0.55 | 0.214 | 0.533 | 0.481 | 0.450 | 0.398 | 0.347 |
|  | 0.65 | 0.281 | 0.524 | 0.569 | 0.609 | 0.642 | 0.523 |
|  | 0.75 | 0.167 | 0.544 | 0.533 | 0.472 | 0.666 | 0.565 |
|  | 0.85 | 0.350 | 0.758 | 0.540 | 0.482 | 0.341 | 0.425 |
|  | 0.95 | 0.600 | 0.477 | 0.267 | 0.661 | 0.400 | 0.514 |

*All value in the Supplemental Table 3 were calculated under the condition (Maternal Characteristics + MAP + UtA-PI+ PLGF + PAPP-A)
