# Supplementary material for: Prediction model of preeclampsia using machine learning based methods: a population based cohort study in China
Source: Front Endocrinol (Lausanne). 2024 Jun 11;15:1345573. doi: 10.3389/fendo.2024.1345573 (PMC11198873; doi:10.3389/fendo.2024.1345573)
Supplement: Supplementary file 4 [file Table_4.docx]

**Supplemental Table 4 Calibration Performance of machine learning algorithms in the preterm PE predictive model**

| **All PE** | **Algorithm** | **Calibration** | | |
| --- | --- | --- | --- | --- |
|  |  | **Brier score**  **(95% CI)** | **Slope**  **(95% CI)** | **Intercept**  **(95% CI)** |
| Maternal Characteristics + MAP + UtA-PI + PLGF + PAPP-A | Logistic Regression | 0.010  [0.009-0.01] | 0.743  [0.699- 0.793] | 0.041  [0.037-0.044] |
|  | Extra Trees Classifier | 0.010  [0.009-0.010] | 0.747  [0.712-0.781] | 0.021  [0.017-0.023] |
|  | Voting Classifier | 0.010  [0.010-0.010] | 0.784  [0.689-0.892] | 0.011  [0.008-0.014] |
|  | Gaussian Process Classifier | 0.011  [0.010-0.011] | 0.327  [0.239-0.419] | 0.130  [0.111-0.148] |
|  | Stacking Classifier | 0.010  [0.010-0.011] | 0.505  [0.203-0.766] | 0.110  [0.040-0.177] |

Brier Score is a measure of the accuracy of probabilistic predictions. The Brier Score ranges from 0 for a perfect model to 1 for the worst model. Lower scores are better; These refer to the calibration slope and intercept when fitting a linear calibration curve to predicted probabilities against observed outcomes. If you were to fit a linear regression to this plot, the slope should ideally be 1, and the intercept should be 0 for a perfectly calibrated model.
