# Supplementary material for: Prediction model of preeclampsia using machine learning based methods: a population based cohort study in China
Source: Front Endocrinol (Lausanne). 2024 Jun 11;15:1345573. doi: 10.3389/fendo.2024.1345573 (PMC11198873; doi:10.3389/fendo.2024.1345573)
Supplement: Supplementary file 5 [file Table_5.docx]

**Supplemental Table 5 Mean model calibration by fold in preterm PE prediction model**

| **(PE-All)** | **Mean predicted value** | **Fraction of positives for fold** | | | | | |
| --- | --- | --- | --- | --- | --- | --- | --- |
| Logistic Regression |  | 1 | 2 | 3 | 4 | 5 | Over All |
|  | 0.05 | 0.008 | 0.032 | 0.008 | 0.008 | 0.008 | 0.008 |
|  | 0.15 | 0.163 | 0.096 | 0.177 | 0.184 | 0.166 | 0.178 |
|  | 0.25 | 0.358 | 0.229 | 0.310 | 0.344 | 0.339 | 0.307 |
|  | 0.35 | 0.320 | 0.381 | 0.318 | 0.366 | 0.410 | 0.335 |
|  | 0.45 | 0.333 | 0.311 | 0.212 | 0.186 | 0.322 | 0.235 |
|  | 0.55 | 0.000 | 0.428 | 0.136 | 0.164 | 0.225 | 0.122 |
|  | 0.65 | 0.503 | 0.569 | 0.258 | 0.125 | 0.233 | 0.245 |
|  | 0.75 | 0.500 | 0.636 | 0.473 | 0.522 | 0.357 | 0.453 |
|  | 0.85 | 0.542 | 0.722 | 0.578 | 0.592 | 0.541 | 0.572 |
|  | 0.95 | 0.501 | 0.804 | 0.619 | 0.542 | 0.586 | 0.603 |
| Extra Trees Classifier |  | 1 | 2 | 3 | 4 | 5 | Over All |
|  | 0.05 | 0.007 | 0.007 | 0.007 | 0.007 | 0.007 | 0.007 |
|  | 0.15 | 0.367 | 0.232 | 0.220 | 0.211 | 0.218 | 0.215 |
|  | 0.25 | 0.321 | 0.317 | 0.314 | 0.311 | 0.313 | 0.317 |
|  | 0.35 | 0.192 | 0.253 | 0.253 | 0.255 | 0.252 | 0.254 |
|  | 0.45 | 0.240 | 0.275 | 0.282 | 0.289 | 0.283 | 0.281 |
|  | 0.55 | 0.500 | 0.507 | 0.487 | 0.535 | 0.495 | 0.498 |
|  | 0.65 | 0.479 | 0.341 | 0.444 | 0.250 | 0.397 | 0.329 |
|  | 0.75 | 0.536 | 0.539 | 0.593 | 0.508 | 0.571 | 0.534 |
|  | 0.85 | 0.592 | 0.600 | 0.664 | 0.565 | 0.639 | 0.595 |
|  | 0.95 | 0.649 | 0.661 | 0.736 | 0.621 | 0.706 | 0.656 |
| Voting Classifier |  | 1 | 2 | 3 | 4 | 5 | Over All |
|  | 0.05 | 0.007 | 0.007 | 0.008 | 0.008 | 0.008 | 0.029 |
|  | 0.15 | 0.061 | 0.069 | 0.074 | 0.077 | 0.076 | 0.094 |
|  | 0.25 | 0.315 | 0.296 | 0.193 | 0.174 | 0.191 | 0.192 |
|  | 0.35 | 0.264 | 0.263 | 0.330 | 0.325 | 0.307 | 0.275 |
|  | 0.45 | 0.277 | 0.399 | 0.357 | 0.368 | 0.345 | 0.363 |
|  | 0.55 | 0.475 | 0.600 | 0.348 | 0.352 | 0.332 | 0.579 |
|  | 0.65 | 0.561 | 0.369 | 0.345 | 0.345 | 0.325 | 0.591 |
|  | 0.75 | 0.647 | 0.333 | 0.464 | 0.544 | 0.399 | 0.561 |
|  | 0.85 | 0.734 | 1.006 | 0.885 | 0.750 | 0.900 | 0.606 |
|  | 0.95 | 0.821 | 0.839 | 0.775 | 0.755 | 0.746 | 0.806 |
| Gaussian Process Classifier |  | 1 | 2 | 3 | 4 | 5 | Over All |
|  | 0.05 | 0.008 | 0.008 | 0.035 | 0.009 | 0.009 | 0.008 |
|  | 0.15 | 0.289 | 0.197 | 0.149 | 0.235 | 0.258 | 0.264 |
|  | 0.25 | 0.225 | 0.269 | 0.225 | 0.263 | 0.269 | 0.331 |
|  | 0.35 | 0.067 | 0.229 | 0.303 | 0.233 | 0.220 | 0.228 |
|  | 0.45 | 0.333 | 0.389 | 0.403 | 0.200 | 0.352 | 0.263 |
|  | 0.55 | 0.167 | 0.167 | 0.468 | 0.200 | 0.282 | 0.200 |
|  | 0.65 | 0.000 | 0.034 | 0.482 | 0.250 | 0.154 | 0.050 |
|  | 0.75 | 0.194 | 0.167 | 0.523 | 0.333 | 0.101 | 0.283 |
|  | 0.85 | 0.167 | 0.515 | 0.652 | 0.167 | 0.325 | 0.525 |
|  | 0.95 | 0.375 | 0.625 | 0.579 | 0.333 | 0.554 | 0.503 |
| Stacking Classifier |  | 1 | 2 | 3 | 4 | 5 | Over All |
|  | 0.05 | 0.009 | 0.034 | 0.009 | 0.008 | 0.009 | 0.008 |
|  | 0.15 | 0.371 | 0.148 | 0.297 | 0.338 | 0.286 | 0.392 |
|  | 0.25 | 0.290 | 0.309 | 0.194 | 0.409 | 0.236 | 0.315 |
|  | 0.35 | 0.083 | 0.344 | 0.154 | 0.260 | 0.086 | 0.110 |
|  | 0.45 | 0.100 | 0.374 | 0.140 | 0.217 | 0.119 | 0.050 |
|  | 0.55 | 0.083 | 0.406 | 0.250 | 0.267 | 0.278 | 0.500 |
|  | 0.65 | 0.125 | 0.472 | 0.500 | 0.000 | 0.750 | 0.391 |
|  | 0.75 | 1.000 | 0.528 | 0.435 | 0.110 | 0.500 | 0.435 |
|  | 0.85 | 0.546 | 0.544 | 0.485 | 0.087 | 0.000 | 0.478 |
|  | 0.95 | 0.606 | 0.415 | 0.534 | 0.000 | 0.448 | 0.521 |

*All value in the Supplemental Table 3 were calculated under the condition (Maternal Characteristics + MAP + UtA-PI+ PLGF + PAPP-A)
