# Supplementary material for: Prediction model of preeclampsia using machine learning based methods: a population based cohort study in China
Source: Front Endocrinol (Lausanne). 2024 Jun 11;15:1345573. doi: 10.3389/fendo.2024.1345573 (PMC11198873; doi:10.3389/fendo.2024.1345573)
Supplement: Supplementary file 6 [file Table_6.docx]

***FMF competing risk model***

The Fetal Medicine Foundation website (fetalmedicine.org) offers a model for screening preeclampsia that calculates risk levels using an Bayesian competing risk algorithm. This algorithm transforms measurements of mean arterial pressure (MAP), uterine artery pulsatility index (UtA-PI), pregnancy-associated plasma protein-A (PAPP-A), and placental growth factor (PLGF), along with factors like gestational age, maternal weight, medical history, and ethnicity into multiples of the median (MoM). After this conversion, the website's algorithm for preeclampsia risk assessment is applied.

**Supplemental Table 6** **Performance of FMF competing risk model for the PE and preterm PE prediction**

| **Type** | **Predictive Factors** | **Model Performance** | | |
| --- | --- | --- | --- | --- |
|  |  | **AUC (95% CI)** | **DR at 5% FPR** | **DR at 10% FPR** |
| All PE | MC | 0.747 [0.712-0.782] | 0.326 | 0.422 |
|  | MC+MAP | 0.815 [0.786-0.844] | 0.396 | 0.513 |
|  | MC+MAP+PAPP-A | 0.825 [0.796-0.853] | 0.404 | 0.539 |
|  | MC+MAP+PAPP-A++UtA-PI | 0.811 [0.782-0.841] | 0.417 | 0.530 |
|  | MC+MAP+PAPP-A++UtA-PI+PLGF | 0.797 [0.767-0.827] | 0.319 | 0.438 |
| Preterm PE | MC | 0.826 [0.767-0.886] | 0.436 | 0.527 |
|  | MC+MAP | 0.851 [0.795-0.907] | 0.455 | 0.600 |
|  | MC+MAP+PAPP-A | 0.879 [0.828-0.931] | 0.527 | 0.691 |
|  | MC+MAP+PAPP-A++UtA-PI | 0.897 [0.852-0.942] | 0.582 | 0.764 |
|  | MC+MAP+PAPP-A++UtA-PI+PLGF | 0.856 [0.799-0.912] | 0.449 | 0.653 |

***Abbreviation:*** DR, Detection rate; FPR, False-positive rate; MC, Maternal Characteristic; MAP, mean arterial pressure; PAPP-A, pregnancy-associated plasma protein-A; PLGF, placental growth factor; UtA-PI, uterine artery pulsatility index.
